# Multivariate Granger Causality Analysis of Acupuncture Effects in Mild Cognitive Impairment Patients: An fMRI Study

**DOI:** 10.1155/2013/127271

**Published:** 2013-08-19

**Authors:** Shangjie Chen, Lijun Bai, Maosheng Xu, Fang Wang, Liang Yin, Xuming Peng, Xinghua Chen, Xuemin Shi

**Affiliations:** ^1^Baoan Hospital, Southern Medical University, Shenzhen 518101, China; ^2^The Key Laboratory of Biomedical Information Engineering of Ministry of Education, Department of Biomedical Engineering, School of Life Science and Technology, Xi'an Jiaotong University, Xi'an 710049, China; ^3^The First Affiliated Hospital, Guangzhou University of Traditional Chinese Medicine, Guangzhou 510405, China; ^4^The First Affiliated Hospital, Tianjin University of Traditional Chinese Medicine, Tianjin 300193, China

## Abstract

Evidence from clinical reports has indicated that acupuncture has a promising effect on mild cognitive impairment (MCI). However, it is still unknown that by what way acupuncture can modulate brain networks involving the MCI. In the current study, multivariate Granger causality analysis (mGCA) was adopted to compare the interregional effective connectivity of brain networks by varying needling depths (deep acupuncture, DA; superficial acupuncture, SA) and at different cognitive states, which were the MCI and healthy control (HC). Results from DA at KI3 in MCI showed that the dorsolateral prefrontal cortex and hippocampus emerged as central hubs and had significant causal influences with each other, but significant in HC for DA. Moreover, only several brain regions had remarkable causal interactions following SA in MCI and even few brain regions following SA in HC. Our results indicated that acupuncture at KI3 at different cognitive states and with varying needling depths may induce distinct reorganizations of effective connectivities of brain networks, and DA at KI3 in MCI can induce the strongest and more extensive effective connectivities related to the therapeutic effect of acupuncture in MCI. The study demonstrated the relatively functional specificity of acupuncture at KI3 in MCI, and needling depths play an important role in acupuncture treatments.

## 1. Introduction

Mild cognitive impairment (MCI) is the key and hot point in cognitive-brain study. Incidence of dementia is widely acknowledged to increase greatly with advancing age. Sousa and coworkers report that dementia made the largest contribution to disability in China, Cuba, Dominican Republic, Mexico, Peru, and urban India [1]. Dementia is a leading cause of death in the United States but is underrecognized as a terminal illness. The median survival was 478 days, and the probability of death within 6 months was 24.7% [2]. Multivariable analyses show that dementia and cognitive impairment are by far the most strongly and independently associated chronic health disorders [3]. Increasing evidence shows that subtle losses in cognitive function may be a symptomatic transition to early AD [4]. MCI is an intermediate state between normal aging and Alzheimer's disease (AD) which is the world's most common dementia [5, 6]. MCI represents a significant risk factor for the development of dementia [4] and is an appropriate condition for investigation [4, 7]. Further research is needed on treatments of delaying the conversion from MCI to AD [4]. However, there is no sufficient evidence that drug can delay long-term progression and conversion to dementia [1, 6]. Feasible complementary and alternative therapies with low side effects, such as acupuncture and exercise, have shown some benefits [8, 9]. The use of acupuncture as a complementary therapeutic way to treat a variety of neurologic diseases, including MCI and AD, is popular in certain parts of the world [10]. In spite of its public acceptance and promising effect, the underlying neural mechanism is still elusive.

Since the late 1990s, functional magnetic resonance imaging (fMRI) has been used to investigate the underlying mechanisms of acupuncture [11], especially the relative functional specificity of acupoints. Neuroimaging studies have indicated that the primary acupuncture effects are mediated by the central nervous system [12–20], and acupuncture can activate certain cognitive-related regions in AD and MCI patients [9]. KI3 is one of the most frequently used acupoint in treatment of cognitive impairment [21]. Our previous studies have also indicated that acupuncture at KI3 can activate certain cognitive-related regions [22–25].

However, there are several problems in fMRI studies of acupuncture. Firstly, most of these studies have been performed on healthy subjects [12–20]. However, acupoint selection often has a very wide range of therapeutic actions related to functional state of the human body based on theory of the traditional chinese medicine (TCM). It is generally accepted that acupuncture plays a homeostatic role and thus may have a greater effect on patients with a pathological imbalance compared with healthy controls (HC) [7, 26, 27]. Therefore, exploring brain function evoked by acupuncture in patients may further help to elucidate its mechanism. In addition, most fMRI studies focus on comparison of brain activity patterns induced by acute effects of acupuncture at acupoint and nonacupoint, or different acupoints. However, few studies have evaluated the modulated effects in the poststimulus resting state networks (RSN) induced by varied needling depth (DA, deep acupuncture; SA, superficial acupuncture) though the depth of needling is also the key of specificity of acupuncture according to the theory of TCM. A primary interest in this area is therefore whether these different depths of needling elicit similar or different responses. Deep acupuncture may better overlap with its proximity to ascending nerve tracks than to the density of cutaneous afferents [28]. In addition, SA has been assumed to minimize the therapeutic effect while triggering most of the nonspecific effects of needling [29]. Therefore, comparing connectivity patterns of brain regions modulated by DA with that of the effects modulated by SA may provide precise and specific modulatory patterns related to the therapeutic effect of acupuncture.

Moreover, many studies generally adopted the block-designed fMRI paradigm conform to the “on-off” specifications. But, function responses induced by acupuncture have time-varying characteristics [15–18]. In recent years, some studies started to pay attention to the sustained effect of the acupuncture and its influence on the postacupuncture RSN with the functional connectivity analysis, which was a kind of undirected graph analysis of temporal correlations between different brain regions [7, 14, 16]. However, little was known about the direction and strength of the information flow between these brain regions modulated by acupuncture. Further investigation of the interregional causal interactions may be helpful to explain the neurophysiological action underlying acupuncture [20, 23]. Recently, a newly multivariate Granger causality analysis (mGCA) has been introduced as an effective connectivity method to analyze direct causal interactions among multiple brain areas from fMRI data [20, 23, 30–33]. By exploring this approach to analyze the causal influences of the activated regions evoked by acupuncture, we can account for its modulatory effects on multiple relevant regions simultaneously. 

Based on the previous study, we employed the mGCA to evaluate the effective connectivity patterns among multiple brain regions following acupuncture at KI3 in MCI patients and HC for DA and SA condition. By examining the directionality and strength of causal influence between multiple brain regions following acupuncture at KI3 in MCI patients and HC for DA and SA condition, we can find whether there is relatively specific modulatory effect at different cognitive states (MCI and HC) and with varying different depths of needling (DA and SA). By detecting the functional specificity of acupuncture for different cognitive states and varied depths of needling during the postresting acupuncture period, we can provide further evidence to explore the relative functional specificity of acupuncture effects.

## 2. Materials and Methods

### 2.1. Subjects

A total of 24 subjects were recruited in the study. 12 MCI patients (1 males and 11 females; ages 59.3 ± 3.3 years; MMSE 26.4 ± 0.9; 7 middle school education and 5 college degree) and 12 age-matched HC subjects (4 males and 8 females; ages 60.6 ± 5.8 years; MMSE 29.8 ± 0.4; 9 middle school education and 3 college degree) were included. All subjects were right handed with normal or corrected-to-normal vision and acupuncture naive according to the edinburgh handedness inventory [34]. MCI patients were diagnosed by using the criteria for amnestic MCI [35], with Mini-Mental State Examination (MMSE) scores >25 [36] and Clinical Dementia Rating (CDR) scale scores of 0.5 [37]. Subjects were excluded if they had serious medical, neurological, or psychiatric illness, or if they were taking medication or other substances known to influence cerebral function, or if they have any contraindications to exposure to a high magnetic field. After being fully explained of the study, all subjects signed the informed consent. All protocols were approved by a local subcommittee on human studies and were conducted in accordance with the Declaration of Helsinki.

### 2.2. Experimental Procedures

**Figure 1:**
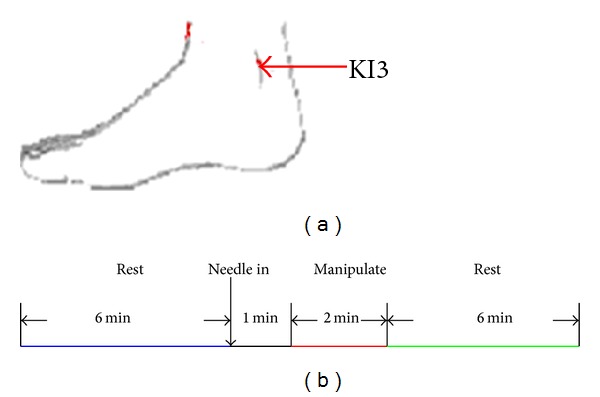
(a) Anatomical locations of the acupuncture stimulation points of KI3. (b) Experimental paradigm. Functional run lasting for 15 min consisted of four phases: 6-minute REST scanning was done before and after acupuncture, inserted in and retained for 1 min and manipulated for 2 min.

For each subject, functional runs lasting for 15 min of the experiment consisted of four phases (Figure 1(b)). In the first phase, a resting state (REST) scan was first conducted for 6 min without any stimulation for a baseline control. In the second phase, an acupuncture needle was then inserted in acupoint KI3 (Taixi, located in a depression between the medial malleolus and heel tendon, Figure 1(a)) on the right leg, and retained for 1 min. In the third phase, the needle was manipulated for 2 min by rotating the needle at a rate of 120 times per min in frequency and at 60° in angle. In the end, another REST scan was conducted for 6 min without any stimulation again. All participants were asked to remain relaxed without engaging in any mental tasks. To facilitate blinding, they were also instructed to keep their eyes closed during fMRI scan to prevent them from actually observing the procedures. According to participants' reports after the scanning, they were affirmed keeping awake during the whole process. Standard acupuncture needles, which are made of ferromagnetic steel, however, are problematic in fMRI studies for several reasons, such as attraction by the scanner's magnetic field, significant image distortions, and signal dropouts, when positioned close to the head or even heating due to absorption of radio frequency (RF). Nonferromagnetic metal needles seem to be the best choice for acupoints outside of the transmitter coil [38]. Therefore, acupuncture stimulation was delivered by a silver needle with 0.35 mm in diameter and 25 mm in length (silver content above 85%, Acupuncture Supplies Company in Suzhou, China) in this study. The acupuncture procedure was conducted by the same experienced and licensed acupuncturist on all subjects. 

We employed two functional runs (DA and SA) for each subject, but only one single stimulation period was given during each of these two runs. The needle was inserted vertically to a depth of 1-2 cm in DA, but of 1-2 mm in SA. Instead of inserting depth, other manipulation methods were all identical in the DA and SA groups. The presentation sequence of two runs was randomized and balanced throughout the population, and every participant performed only one run each week in order to eliminate potential long-lasting effect following acupuncture administration. All participants were not informed of the order in which these runs would be performed. 

At the end of each acupuncture scan, the subjects were questioned about aching, pressure, soreness, heaviness, fullness, warmth, coolness, numbness, tingling, dull or sharp pain, and any other sensations they felt during the stimulation [15, 20, 39]. A 10-point visual analogue scale (VAS), which was scaled at 0 = no sensation, 1–3 = mild, 4–6 = moderate, 7-8 = strong, 9 = severe, and 10 = unbearable sensation, was adopted to self-rate the intensities about the deqi sensations [15, 39, 40]. To quantitatively summarize the full multivariate breadth and depth of the De-qi sensations for each subject, the VAS index was calculated [15, 20, 39].

### 2.3. fMRI-Scanning Procedure

Magnetic resonance imaging data were collected from a 3T MR scanner. A standard birdcage head coil was used, along with restraining foam pads to minimize head motion and to diminish scanner noise. For each subject, functional scans of acupuncture stimulation were taken after the anatomical scans. The scan covered the entire brain including the cerebellum and brainstem. Thirty axial slices were obtained using a T2*-weighted single-shot, gradient-recalled echo planar imaging (EPI) sequence (TR = 2000 ms, TE = 30 ms, FOV = 220  mm  ×  220 mm, matrix = 64  ×  64, thickness = 4 mm, Slice Space = 1 mm, flip angle = 77°). High-resolution structural information on each subject was also acquired using three-dimensional (3D) MRI sequences with a voxel size of 1 mm^3^ for anatomical localization (TR = 2.1 s, TE = 4.6 ms, FOV = 230 mm  ×  230 mm, matrix = 256  ×  256, slice thickness = 1 mm, flip angle = 8°).

### 2.4. fMRI Data Analysis

Preprocessing was performed using the Statistical Parametric Mapping software (SPM5, http://www.fil.ion.ucl.ac.uk/spm/). Initially, the first five time points were discarded in order to avoid the instability of the initial MRI signal [40]. The image data underwent slice-timing correction and realignment for head motions using least-squares minimization. None of the subjects had head movements exceeding 1 mm on any axis and head rotation greater than one degree. A mean image created from the realigned volumes was co-registered with the subject's individual structural T1-weighted volume image [20]. Then, the images were normalized to the standard EPI template and re-sampled to a voxel size of 2 mm  ×  2 mm ×  2 mm [41]. Subsequently, these data were filtered by using a bandpass filter (0.01–0.08 Hz) to reduce the effect of low-frequency drift and high-frequency noise [42, 43]. Finally, the images were smoothed spatially by using a 6 mm full-width-at-half maximum (FWHM) isotropic Gaussian kernel.

Taking into account of the sustained effects of acupuncture, the resting period before acupuncture was taken as the baseline. For each subject, the difference in the BOLD response was estimated at every voxel across the whole brain by using the general linear model (GLM) in SPM5. The obtained *t*-maps at individual levels were then entered into the “random effect” group analysis framework by the one-sample *t*-test summary statistic (*P* < 0.005, uncorrected). The statistical maps indicated the brain activation in response to acute effects of acupuncture, thereby functionally defining ROI. Each peak voxel with its nearest 10 neighbors was defined as a group ROI. Considering the anatomical variance across subjects, subject-specific peak voxels and subject-specific ROIs were defined on individual *t*-maps as follows. The given group ROI was used as a mask and then, based on individual *t*-maps, and the voxel with the largest *t*-value within this mask served as the subject-specific peak voxel. ROIs were selected based on the acupuncture-stimulation results. Firstly, the time series from the poststimulus resting period of BOLD signal intensities from these selected ROIs were selected. Then, the time series were averaged across voxels within each ROI and normalized across subjects separately for each group to form a single vector per ROI [20].

In order to describe the effective connectivity during the postacupuncture resting period [30], the mGCA was used to detect causal interactions between brain regions by computing directed transfer function (DTF) from a multivariate autoregressive model of the time course of selected ROIs [20, 23]. Based on the principle of Granger causality, the DTF was rendered in a multivariate formulation [44]. Therefore, the DTF can effectively model the inherently multivariate nature of neuronal networks. The algorithm was coded in MATLAB7 (The MathWorks, Inc.) [20]. Effective connectivity graphs were constructed using the thickness of connecting lines and arrows to indicate the strength and direction of the causal influences. Only links that showed significant effective connectivity were presented in the network (*P* < 0.05). Graphs were visualized using Pajek software (http://vlado.fmf.uni-lj.si/pub/networks/pajek/).

## 3. Results

### 3.1. Psychophysical Response

**Figure 2:**
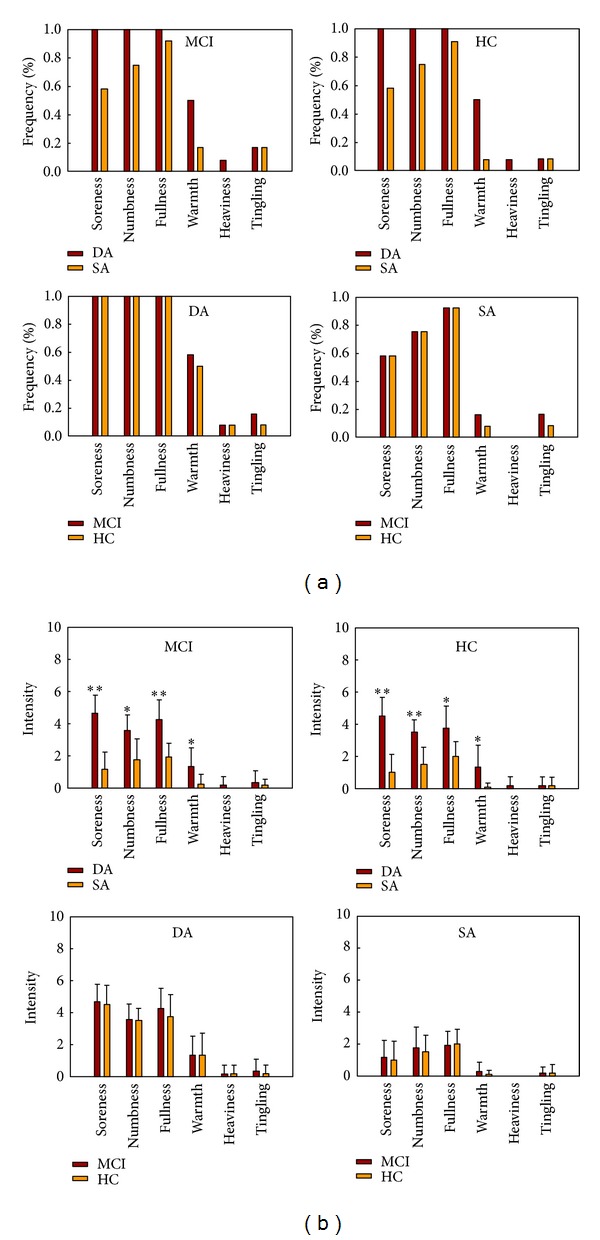
(a) The prevalence of deqi sensations. It was expressed as the percentage of the individuals in the group that reported the sensation (at least one subject experienced the seven sensations listed). (b) The intensity of sensations. It was expressed as the average score ± S.E by measuring on a scale from 0 denoting no sensation to 10 denoting an unbearable sensation. The intensity of numbness, fullness, and soreness was found to be greater for the DA than the SA under Fisher's Exact Test (*P < 0.01; **P < 0.001).

The prevalence of deqi sensations was expressed as the percentage of the individuals in the group that reported the sensations (Figure 2(a)). Differences did exist with respect to the type of sensations. Both in the MCI and HC group, the soreness, numbness, fullness, warmth, and heaviness were found to be more frequent for DA than that of SA. Whether for the DA or SA, warmth and tingling were found to be more frequent in the MCI group than HC group. 

The intensity of sensations was expressed as the average score ± S.E. (Figure 2(b)). Differences did also exist with respect to the type of sensations. In both MCI and HC groups, the sensations of soreness, numbness, fullness, and warmth were found to be stronger for DA than SA. For both conditions, a statistical analysis found no significant difference between the MCI and HC groups with regard to the intensity of these sensations.

### 3.2. mGCA Result of Resting Brain Networks Modulated by Acupuncture

**Figure 3:**
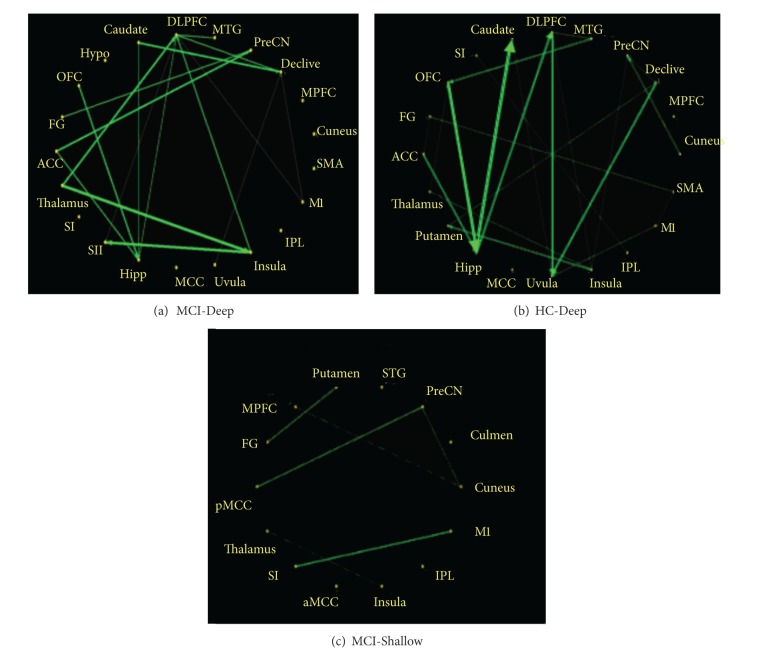
Multivariate Granger causality relationships. There were remarkably causal interactions (*P* < 0.05) following acupuncture at KI3 in MCI-Deep, HC-Deep, and MCI-Shallow, but no causal interactions following acupuncture at KI3 in HC-Shallow. Relative strength of path weights (in arbitrary units) was indicated by the width of the arrows. Abbreviations: ACC, anterior cingulate cortex; FG, fusiform gyrus; OFC, orbitofrontal cortex; HYPO, hypothalamus; DLPFC, dorsolateral prefrontal cortex; MTG, middle temporal gyrus; PreCN, precuneus; MPFC, medial prefrontal cortex; SMA, supplementary motor area; M1, primary motor cortex; IPL, inferior parietal lobule; MCC, middle cingulate cortex; HIPP, hippocampus; SII, secondary somatosensory cortex; SI, primary somatosensory cortex; pMCC, middle part of the posterior cingulate cortex; STG, superior temporal gyrus; aMCC, middle part of the anterior cingulate cortex.

In this study, we explored the causal interactions within and among the resting brain networks modulated by acupuncture at KI3 in MCI and HC, for DA and SA. The effective connectivity patterns of resting brain networks were described as directed graphs. The thickness of connecting lines and the directions of arrows indicated strength and directions of the causal influences (green line in Figure 3). Only significant effective connectivity (*P* < 0.05) was presented in the graphs. 

Following acupuncture at KI3 in MCI-Deep, the mGCA result showed that the dorsolateral prefrontal cortex (DLPFC) and hippocampus (Hipp) emerged as central hubs. The DLPFC received causal inflows from most nodes in the brain network, including the Thalamus, Insula, middle temporal gyrus (MTG), and primary motor cortex (M1). The Hipp received causal inflows from the DLPFC, anterior cingulate cortex (ACC), orbitofrontal cortex (OFC), and Caudate. In addition, Insula received causal inflows from Thalamus. The precuneus (PreCN) received causal inflows from ACC and fusiform gyrus (FG). The secondary somatosensory cortex (SII) received causal inflows from Insula and DLPFC. Declive received causal inflows from Caudate, DLPFC, M1, and Uvula. There were strong causal inflows from Thalamus to Insula and DLPFC, from Insula to SII and DLPFC, from Caudate to Declive, from OFC to Hipp, and from ACC to PreCN and Hipp. Of interests, we found that the DLPFC and Hipp were not only central hubs but also had significant causal influence on each other. The path weights of mGCA result for MCI-Deep were tabulated in Table 1 with significant connections shown in blue color.

Notably, several of these brain regions (the Hipp and DLPFC) mentioned above also have remarkably causal interactions following acupuncture at KI3 in HC-Deep, but they were more noncohesive than in MCI-Deep. Following acupuncture at KI3 in HC-Deep, the mGCA result showed that the Hipp, OFC, DLPFC, and Uvula emerged as central hubs. There were strong causal inflows from ACC and OFC to Hipp, from DLPFC and Declive to Uvula, from MTG to OFC, from Cuneus to PreCN, from Insula to DLPFC and Putamen, from Hipp to Caudate and DLPFC, and from Thalamus to OFC and Insula. The path weights of mGCA result for HC-Deep were tabulated in Table 2 with significant connections shown in blue color.

Only several of brain regions had remarkably causal interactions following acupuncture at KI3 in MCI-Shallow. Following acupuncture at KI3 in MCI-Shallow, the mGCA result showed that there were causal inflows from pMCC and Cuneus to PreCN, from MPFC to Cuneus, from Putamen to FG, from M1 to primary somatosensory cortex (SI), and from Insula to Thalamus. The path weights of mGCA result for MCI-shallow were tabulated in Table 3 with significant connections shown in blue color. It is also notable that a few brain regions had remarkably causal interactions following acupuncture at KI3 in HC-Shallow.

## 4. Discussion

KI3 is one of the most frequently used acupoints and prove to have various efficacies in the treatment of dementia [15]. Our previous studies have indicated that acupuncture at KI3 can activate certain cognitive-related regions [22–25]. In this study, we have further investigation on effective connectivity of postacupuncture resting brain networks at KI3 in different cognitive states and with varying acupuncture depths.

Previous functional connectivity analysis primarily focused on the correlation patterns, and this method was limited to assess brain regions functionally connected to the initially selected seed and was unable to directly characterize interactions between multiple brain regions [20]. Few studies paid attention to the direction and strength of the information flow between these brain regions modulated by acupuncture. Effective connectivity in the poststimulus resting brain may underlie the neural mechanism of acupuncture for the treatment of MCI, but very few studies have yet investigated it. By visualizing the effective connectivity, we can obtain both the direction and strength of the information flow between multiple brain regions in the resting state network following acupuncture. In this study, a newly mGCA was employed to explore the specific effective connectivity poststimulus resting period following acupuncture at KI3. Our results demonstrated that acupuncture at KI3 in different cognitive states and with varying acupuncture depths may exert heterogeneous modulatory effects on the causal interactions of brain areas during the poststimulus resting state. These different effective connectivity patterns may be related to the special effects of acupuncture in clinical settings [20, 23]. Our findings may be helpful to understand the basic neurophysiological mechanisms underlying the specificity of acupuncture.

According to the mGCA results following acupuncture at KI3 in MCI-Deep, we identified that brain regions have extensive causal interactions, mainly locating at the DLPFC, Hipp, Thalamus, Insula, Declive, MTG, ACC, OFC, and Caudate. The results from mGCA showed that the DLPFC and Hipp emerged as central hubs and had significant causal influence on each other. The DLPFC received causal inflows from most nodes in the brain network, including the Thalamus, Insula, MTG, and M1. One study showed that the DLPFC disconnections may be the substrates of cognitive impairments in MCI patients [45]. The DLPFC plays a role in sustaining attention and working memory [46, 47]. Lesions in the DLPFC can impair the short-term memory and cause difficulties in inhibiting responses [46]. In addition, the DLPFC has recently been found to be involved in exhibiting self-control [47]. The inhibition of the right DLPFC could modulate the excitability of a network of brain regions, in the ipsilateral as well as in the contralateral hemisphere, to enhance function in HC or restore an adaptive equilibrium in the MCI [48]. In addition, the Hipp received causal inflows from the DLPFC, ACC, OFC, and Caudate. In addition, the Insula received strong causal inflows from the Thalamus. One study showed abnormalities in the connectivity associated with the hippocampus in MCI [49]. Functional results indicated that the hippocampus reduced cortical activation in the DMN for MCI patients, compared with age- and education-matched healthy elderly controls [50]. The hippocampus plays a key role in a distributed network supporting memory encoding and retrieval [51]. The meta-analyses of 1,768 functional neuroimaging experiments revealed four functionally distinct regions on the human insula, which map to the social-emotional, the sensorimotor, the olfactory-gustatory, and the cognitive network of the brain [52]. Abnormal insula functional network is associated with episodic memory decline in amnestic mild cognitive impairment [53]. The thalamus is functionally connected to the hippocampus as part of the extended hippocampal system [54]. The literature seems to support the hypothesis that specialization of cortical areas in the MTL, as for their involvement in recollection and familiarity processes, may also extend to discrete regions of the thalamus [55]. Functional connectivity between the left thalamus and a set of regions was decreased in MCI, increased functional connectivity between the left thalamus and the right thalamus in MCI [56]. The DLPFC connectivity with the IPL and thalamus significantly correlated with the cognitive performance of patients measured by minimental state examination, clock drawing test, and California verbal learning test scores [57]. Therefore, the causal interactions related with these cognitive-related regions following DA may relate to the therapeutic effect of acupuncture for the treatment of MCI. 

In addition, we also found that the hippocampus and DLPFC related with cognitive-related regions mentioned above also have remarkably causal interactions following acupuncture at KI3 in HC-Deep. Compared with that in MCI, causal interactions were significant noncohesive in HC. Many studies showed that there were specific functional changes of brain in the MCI patients compared with age- and education-matched healthy elderly controls [45, 50]. Acupuncture plays a homeostatic role and thus may have a greater effect on patients with a pathological imbalance compared to HC [7, 26, 27]. For HC, effect of acupuncture is weak. Our results demonstrated that there were different effective connectivities of postacupuncture at KI3 in different cognitive states, and effect of acupuncture was stronger in MCI compared with that in HC.

Moreover, only several of brain regions had remarkably causal interactions following acupuncture at MCI-Shallow. Most interestingly, no brain regions had remarkably causal interactions following acupuncture at HC-Shallow. The mGCA results demonstrated that effective connectivity of postacupuncture at KI3 in shallow of needling was very weak. Furthermore, the effect of postacupuncture at KI3 in HC-Shallow of needling was too weak to evoke causal interactions. Deep insertion of the needle affects several structures including the skin, muscle fascia, and muscle, and acupoints may better overlap with their proximity to ascending nerve tracks than to the density of cutaneous afferents [28]. The muscular afferents affect greater number of receptors to achieve a special clinical effect than the cutaneous afferents from shallow insertion of the needle. Therefore, the stronger effective connectivity associated with the cognitive-related regions following DA may suggest that deep muscle acupuncture has a better therapeutic effect for the treatment of MCI. The heterogeneous effective connectivity patterns between DA and SA may suggest the importance of the muscular afferents in acupuncture. The results of psychophysical response showed that there were different prevalence and intensity of sensations for both conditions. Soreness, numbness, fullness, and warmth were found to be more frequent and stronger for DA than SA. Effect of acupuncture is better if sensation is stronger based on TCM. But, one study suggested that acupuncture needle stimulation at two different depths of needling, superficial, and deep did not elicit significantly different BOLD responses [58]. This result may be related to the mistaken design of block design and acupuncture in HC. Moreover, the author also pointed out that the participants in that study were healthy individuals, and it is possible that superficial and deep acupuncture could potentially have different effects when being used to treat people with pathology. Our results demonstrated that there was different effective connectivity of postacupuncture at KI3 in different depths of needling, and effect of acupuncture was stronger for DA compared with that for SA.

In conclusion, results indicated that acupuncture at acupoint KI3 in different cognitive state and different acupuncture depth may induce distinct reorganization of the effective connectivity across different neural subsystems, and acupuncture at KI3 in MCI-Deep can induce the strongest and more extensive effective connectivity related to the therapeutic effect of acupuncture for the treatment of MCI. 

## 5. Conclusions

The current study demonstrated that the significantly enhanced correlations in the cognitive-related brain regions following acupuncture may be related to the therapeutic effects of acupuncture for the treatment of MCI. Our results also revealed that there existed more tightly effective connectivity patterns during the poststimulus resting state following acupuncture at acupoint KI3 in MCI patients compared to HC, stronger effective connectivity patterns for DA compared to SA, and acupuncture effects could last for a long period even though the needling process was terminated. We suggested that the distinct modulation patterns of the resting brain networks attributed to the specific effects which was evoked by acupuncture in different cognitive states and different needling depths. The study demonstrated that acupoint can play a better role in the suitable depth of needle and disease state. This preliminary finding may provide a new clue to decipher the relatively functional specificity of acupuncture effects. Our findings may help to understand the neurophysiological mechanism underlying acupuncture specificity and to employ KI3 for the treatment of MCI in the clinical practice.

## Figures and Tables

**Table 1:** Path weights from multivariate Granger causality analyses under the MCI-Deep (*P* < 0.01).

	ACC	FG	OFC	Hypo	Caudate	DLPFC	MTG	PreCN	Declive	MPFC	Cuneus	SMA	M1	IPL	Insula	Uvula	MCC	Hipp	SII	SI	Thalamus
ACC	—	×	×	×	×	×	×	**2.03**	×	×	×	×	×	×	×	×	×	**1.56**	×	×	×
FG	×	—	×	×	×	×	×	**0.79**	×	×	×	×	×	×	×	×	×	×	×	×	×
OFC	×	×	—	×	×	×	×	×	×	×	×	×	×	×	×	×	×	**2.15**	×	×	×
Hypo	×	×	×	—	×	×	×	×	×	×	×	×	×	×	×	×	×	×	×	×	×
Caudate	×	×	×	×	—	×	×	×	**1.97**	×	×	×	×	×	×	×	×	**0.78**	×	×	×
DLPFC	×	×	×	×	×	—	×	×	**1.1**	×	×	×	×	×	×	×	×	**0.98**	**0.43**	×	×
MTG	×	×	×	×	×	**1.1**	—	×	×	×	×	×	×	×	×	×	×	×	×	×	×
PreCN	×	×	×	×	×	×	×	—	×	×	×	×	×	×	×	×	×	×	×	×	×
Declive	×	×	×	×	×	×	×	×	—	×	×	×	×	×	×	×	×	×	×	×	×
MPFC	×	×	×	×	×	×	×	×	×	—	×	×	×	×	×	×	×	×	×	×	×
Cuneus	×	×	×	×	×	×	×	×	×	×	—	×	×	×	×	×	×	×	×	×	×
SMA	×	×	×	×	×	×	×	×	×	×	×	—	×	×	×	×	×	×	×	×	×
M1	×	×	×	×	×	**0.57**	×	×	**0.67**	×	×	×	—	×	×	×	×	×	×	×	×
IPL	×	×	×	×	×	×	×	×	×	×	×	×	×	—	×	×	×	×	×	×	×
Insula	×	×	×	×	×	**1.56**	×	×	×	×	×	×	×	×	—	×	×	×	**3.2**	×	×
Uvula	×	×	×	×	×	×	×	×	**0.43**	×	×	×	×	×	×	—	×	×	×	×	×
MCC	×	×	×	×	×	×	×	×	×	×	×	×	×	×	×	×	—	×	×	×	×
Hipp	×	×	×	×	×	×	×	×	×	×	×	×	×	×	×	×		—	×	×	×
SII	×	×	×	×	×	×	×	×	×	×	×	×	×	×	×	×	×	×	—	×	×
SI	×	×	×	×	×	×	×	×	×	×	×	×	×	×	×	×	×	×	×	—	×
Thalamus	×	×	×	×	×	**2.34**	×	×	×	×	×	×	×	×	**3.45**	×	×	×	×	×	—

**Only the significant paths are listed**. Influences are from column ROI to row ROI. × indicates the weights below the significance. Abbreviations: ACC: anterior cingulate cortex; FG: fusiform gyrus; OFC: orbitofrontal cortex; HYPO: hypothalamus; DLPFC: dorsolateral prefrontal cortex; MTG: middle temporal gyrus; PreCN: precuneus; MPFC: medial prefrontal cortex; SMA: supplementary motor area; M1: primary motor cortex; IPL: inferior parietal lobule; MCC: middle cingulate cortex; HIPP: hippocampus; SII: secondary somatosensory cortex; SI: primary somatosensory cortex.

**Table 2:** Path weights from multivariate Granger causality analyses under the HC-Deep (*P* < 0.01).

	ACC	FG	OFC	SI	Caudate	DLPFC	MTG	PreCN	Declive	MPFC	Cuneus	SMA	M1	IPL	Insula	Uvula	MCC	Hipp	Putamen	Thalamus
ACC	—	**0.66**	×	×	×	×	×	×	×	×	×	×	×	×	×	×	×	**1.92**	×	×
FG	×	—	×	×	×	×	×	×	×	×	×	×	×	×	×	×	×	×	×	×
OFC	×	×	—	×	×	×	×	×	×	×	×	×	×	×	×	×	×	**3.45**	×	×
SI	×	×	×	—	×	×	×	×	×	×	×	×	×	**0.4**	×	×	×	×	×	×
Caudate	×	×	×	×	—	×	×	×	×	×	×	×	×	×	×	**0.46**	×	×	×	×
DLPFC	×	×	×	×	×	—	**0.4**	×	×	×	×	×	×	×	×	**2.1**	×	×	×	×
MTG	×	×	**2.04**		×	×	—	×	×	×	×	×	×	×	×	×	×	×	×	×
PreCN	×	×	×	×	×	×	×	—	×	×	×	×	×	×	×	×	×	×	×	×
Declive	×	×	×	×	×	×	×	**0.94**	—	×	×	×	×	×	×	**2.08**	×	×	×	×
MPFC	×	×	×	×	×	×	×	×	×	—	×	×	×	×	×	×	×	×	×	×
Cuneus	×	×	×	×	×	×	×	**1.29**	×	×	—	×	×	×	×	×	×	×	×	×
SMA	×	**0.26**	×	×	×	×	×	×	×	×	×	—	**0.3**	×	×	×	×	×	×	×
M1	×	×	×	×	×	×	×	×	×	×	×	×	—	×	×	**0.87**	×	×	×	×
IPL	×	×	×	×	×	×	×	×	×	×	×	×	×	—	×	×	×	×	×	×
Insula	×	×	×	×	×	**1.12**	×	**0.48**	×	×	×	×	×	×	—	×	×	×	**2.2**	×
Uvula	×	×	×	×	×	×	×	×	×	×	×	×	×	×	×	—	×	×	×	×
MCC	×	×	×	×	×	×	×	×	×	×	×	×	×	×	×	×	—	×	×	×
Hipp	×	×	×	×	**3.78**	**1.56**	×	×	×	×	×	×	×	×	×	×	×	—	×	×
Putamen	×	×	×	×	×	×	×	×	**0.83**	×	×	×	×	×	×	×	×	×	—	×
Thalamus	×	×	**2.34**	×	×	×	×	×	×	×	×	×	×	×	**1.45**	×	×	×	×	—

**Only the significant paths are listed**. Influences are from column ROI to row ROI. × indicates the weights below the significance. Abbreviations: ACC: anterior cingulate cortex; FG: fusiform gyrus; OFC: orbitofrontal cortex; SI: primary somatosensory cortex; DLPFC: dorsolateral prefrontal cortex; MTG: middle temporal gyrus; PreCN: precuneus; MPFC: medial prefrontal cortex; SMA: supplementary motor area; M1: primary motor cortex; IPL: inferior parietal lobule; MCC: middle cingulate cortex; HIPP: hippocampus.

**Table 3:** Path weights from multivariate Granger causality analyses under the MCI-Shallow (*P* < 0.01).

	pMCC	FG	MPFC	Putamen	STG	PreCN	Culmen	Cuneus	M1	IPL	Insula	aMCC	SI	Thalamus
pMCC	—	×	×	×	×	**0.98**	×	×	×	×	×	×	×	×
FG	×	—	×	×	×	×	×	×	×	×	×	×	×	×
MPFC	×	×	—	×	×	×	×	**0.63**	×	×	×	×	×	×
Putamen	×	**0.78**	×	—	×	×	×	×	×	×	×	×	×	×
STG	×	×	×	×	—	×	×	×	×	×	×	×	×	×
PreCN	×	×	×	×	×	—	×	×	×	×	×	×	×	×
Culmen	×	×	×	×	×	×	—		×	×	×	×	×	×
Cuneus	×	×	×	×	×	**0.56**	×	—	×	×	×	×	×	×
M1	×	×	×	×	×	×	×	×	—	×	×	×	**1.34**	×
IPL	×	×	×	×	×	×	×	×	×	—	×	×	×	×
Insula	×	×	×	×	×	×	×	×	×	×	—	×	×	**0.32**
aMCC	×	×	×	×	×	×	×	×	×	×	×	—	×	×
SI	×	×	×	×	×	×	×	×	×	×	×	×	—	×
Thalamus	×	×	×	×	×	×	×	×	×	×	×	×	×	—

**Only the significant paths are listed**. Influences are from column ROI to row ROI. × indicates the weights below the significance. Abbreviations: pMCC: posterior middle cingulate cortex; FG: fusiform gyrus; MPFC: medial prefrontal cortex; STG: superior temporal gyrus; PreCN: precuneus; M1: primary motor cortex; IPL: inferior parietal lobule; aMCC: anterior middle cingulate cortex; SI: primary somatosensory cortex.
